# HER2 as a Promising Target for Cytotoxicity T Cells in Human Melanoma Therapy

**DOI:** 10.1371/journal.pone.0073261

**Published:** 2013-08-27

**Authors:** Juan Ma, Huamin Han, Deruo Liu, Wei li, Hongxiang Feng, Xin Xue, Xiaoran Wu, Ge Niu, Ge Zhang, Yunfeng Zhao, Changzhen Liu, Hua Tao, Bin Gao

**Affiliations:** 1 CAS Key Laboratory of Pathogenic Microbiology and Immunology, Institute of Microbiology, Chinese Academy of Sciences, Beijing, P. R. China; 2 Key Laboratory of Infection and Immunity, Institute of Biophysics, Chinese Academy of Sciences, Beijing, P. R. China; 3 Department of Thoracic Surgery, China-Japan Friendship Hospital, Beijing, P. R. China; 4 Department of Immunology, Basic Medical Theory of Chinese Medicine, China Academy of Chinese Medical Sciences, Beijing, P. R. China; 5 China-Japan Joint Laboratory of Molecular Immunology and Microbiology, Institute of Microbiology, Chinese Academy of Sciences, Beijing, P. R. China; Istituto Superiore di Sanità, Italy

## Abstract

Anti-HER2/neu antibody therapy has been reported to mediate tumor regression of HER2/ neu^+^ tumors. Here we demonstrated the expression of HER2 in a wide range of human melanoma cells including a primary culture and seven cell lines, and we further investigated whether HER2 could be served as a target for T cell mediated immunotherapy of human melanoma. Specific cytolytic activity of activated T cells (ATC) armed with anti-CD3 x anti-HER2 bispecific antibody (HER2Bi-Ab) against Malme-3M-luc cells was evaluated by bioluminescent signal generated by luciferase reporter which did not alter HER2 expression or proliferation ability of Malme-3M cells. Contrast with unarmed ATC, increased cytotoxic activity of HER2Bi-armed ATC against Malme-3M-luc cells was observed at effector/target (E/T) ratios of 1:1, 5:1, and 20:1. Moreover, HER2Bi-armed ATC expressed higher level of activation marker CD69 and secreted significantly higher level of IFN-γ than unarmed ATC counterpart at the E/T ratio of 20:1. In addition, compared with anti-HER2 mAb (Herceptin®) or unarmed ATC, HER2Bi-armed ATC showed remarkable suppression effect on Malme-3M-luc tumor cells. Furthermore, in melanoma tumor cell xenograft mice, infusion of HER2Bi-armed ATC successfully inhibited the growth of melanoma tumors. The anti-tumor effect of HER2Bi-armed ATC may provide a promising immunotherapy for melanoma in the future.

## Introduction

Melanoma is an increasingly common and potentially dangerous type of skin and mucosal cancer associated with a poor prognosis. Surgery, radiotherapy and chemotherapy are traditional strategies for melanoma, but the control for metastasis is difficult, and only 10% of metastatic melanoma patients survive more than 5 years [1]. Innovative and more effective therapies for melanoma are on-going. Immunotherapies including vaccination and adoptive T cell therapy hold great promise [2], both of which have been targeted to tumor associated antigens such as MART-1, gp100, tyrosinase [3], MAGE family, BAGE, GAGE and gp75 [4,5]. Immunosuppressive molecule CD200 and immune checkpoint proteins such as CTLA-4, PD-1 and CD40 expressed on melanoma cells have also been identified as possible immunotherapy candidates [6]. The development of antibodies and small molecules that either inhibit or promote their activity has lent a huge impetus to the immunotherapy of melanoma [7]. Via blocking the CTLA-4 inhibitory signal, and allowing cytotoxic T lymphocytes (CTL) to destroy tumor cells [8], ipilimumab was approved in 2011 by FDA for the treatment of melanoma.

The HER2/neu gene, also known as cerbB2, encodes a 185-kDa transmembrane glycoprotein, HER2. The protein belongs to the family of epidermal growth factor receptor, an oncoprotein with intrinsic tyrosine kinase activity. HER2 overexpression has been detected in many human tumor types, including breast, ovarian, endometrial, salivary gland, gastric, bladder and pancreatic cancers [9–13]. Its expression is normally associated with poor clinical outcome [14] even at a very low level. The use of Herceptin®, a humanized monoclonal antibody that binds the extracellular, juxtamembrane domain of HER2, has been proven to be an effective treatment for breast cancer in which HER2 overexpression is present [15,16].

Although some investigators argued that HER2/neu expression was rare in metastatic and advanced melanoma [17–21], many investigators demonstrated the presence of HER2/neu expression during melanoma progression and metastases contrast to normal melanocytes [22,23]. Bodey et al. [24] reported that increased expression of HER2 appeared in 8 out of 10 patients with metastatic melanoma. Incidence of HER2 expression in patients with thick cutaneous primary melanoma was similar to that reported in breast cancer. Therefore, the success of Herceptin® in the treatment of breast cancer suggests its potential role in the treatment of melanoma expressing HER2, although some evidence suggests that therapy specifically targeting HER2 may not provide the benefit for patients with metastatic melanoma or as an adjuvant therapy for melanoma patients at high risk for recurrence [19].

In this study we demonstrated that HER2 could be served as a target for immunotherapy of human melanoma after confirmation of the expression of HER2 in human melanoma cells.

## Materials and Methods

### Ethic Statement

This study and experimental protocols involved in animals were approved by Biomedical Research Ethics Committee of CAS Key Laboratory of Pathogenic Microbiology and Immunology.

### 1: Cell lines

The following cell lines were cultured in RPMI 1640 (GIBCO): a primary human melanoma cell culture, OCM-1, OMM-1, and 92-1 human melanoma [25], K562 human leukemia (obtained from ATCC), and B16-luc cell line (from Shanghai Genomic s Inc.). Human melanoma cell line Malme-3M, Mel 624, Mel 888 and SK Mel28 (obtained from ATCC) were cultured in DMEM (GIBCO). Media were supplemented with 10% FCS, 100 U/ml penicillin, 100 μg/ml streptomycin.

### 2: Vector construction

The luciferase gene was amplified used the following primes: luc2-*Nhe*I: 5’- ACAATTGCTAGCATGGAAGACGCCAAAAAC-3’, luc2-*EcoR*I:5’-GACAGAAT TCTTACACGGCGATCTTTCC-3’, the fragment was inserted into the vector 3.7lnGFP (lentivector previously constructed in our lab [26]) to replace GFP at the *Nhe*I and *EcoR*I sites to make 3.7lnluc2, which contained a BirA substrate peptide linked to a truncated form of human low-affinity nerve growth factor receptor (△LNGFR). Another 3.7BirA plasmid (previously constructed in our lab) contained a biotin ligase BirA, and biotinylated △LNGFR was transported to the cell surface where it labeled the target cells.

### 3: Generation of Malme-3M-luc reporter cell lines

The lentiviral transfer vector 3.7lnluc2 and 3.7BirA are both self-inactivating lentivector. VSV-G pseudotyped lentiviral vectors stocks were prepared by calcium phosphate-based transfection of 293T cells with 6 μg of packaging plasmids pLP1, pLP2 and pLP/VSVG (Invitrogen, Carlsbad, CA, USA) respectively and 3 μg of transfer vector respectively. The supernatant was filtered through a 0.45 μm filter and frozen in aliquots at -80°C until use. The viruses were used to transduce Malme-3M cells in the presence of 4 μg/ml polybrene at an MOI of 1 by spin inoculation in a centrifuge at 1,800 x g for 90 minutes at 32°C in 6-well tissue culture plates and incubated at 37°C overnight, fresh medium was added after the overnight incubation. This process was repeated once after every 24 hours later. Magnetic bead-based cell separation was performed 72 hours post-infection as the protocol published previously [26]. After lentiviral infection and separation of transduced cell lines, the transduction efficiency was monitored by flow cytometry. The bioluminescence imaging signals of Malme-3M-luc reporter cell lines were measured with the Xenogen IVIS system on serial dilution of Malme-3m-luc cells seeded in a black 96-well plate in PBS containing D-luciferin substrate at final concentration of 0.15 mg/ml.

### 4: Preparation and cryopreservation of activated T cells (ATC) from isolated peripheral blood lymphocytes

Peripheral mononuclear blood cells (PBMCs) were isolated using Ficoll density gradient centrifugation from healthy donors supplied by Beijing Blood Bank. Anti-CD3 activated ATC were expanded 14 days from PBMCs as the method previously described [27]. On day 15, ATC expansion products of donors were averaged 98.85 ± 1.06% CD3^+^ cells (38.4± 18.1% CD4^+^ and 66.3 ± 9.83% CD8^+^). ATC were cultured in RPMI1640 supplemented with FCS and cryopreserved. The project was carried out according to the protocols approved by the Biomedical Research Ethics Committee of CAS Key Laboratory of Pathogenic Microbiology and Immunology.

### 5: Synthesis of anti-CD3 × anti-Her2 Bispecific Antibody (HER2Bi-Ab) and arming of ATC

6 mg/ml of Anti-HER2 (Herceptin®, Roche) was activated with 10 fold molar excess of sulfo-SMCC (1.9 mg/ml) firstly. 6 mg/ml of Anti-CD3 (OKT3, eBioscience, San Diego, CA, USA) was activated with 10 fold molar excess of Traut’s reagent (2.2 mg/ml). Both reaction mixtures were incubated at room temperature for 1 hour and excess crosslinker was removed using PD-10 column. The cross-linked mAbs were mixed immediately at equimolar ratios and hetero conjugated at 4°C overnight. Cryopreserved ATC was thawed, washed, counted, and armed with HER2Bi-Ab at a concentration of 50 ng/10^6^ cells at room temperature for 15 minutes followed by washing the cells to eliminate unbound antibodies.

### 6: In vitro cell proliferation assay

Melanoma cells were seeded (2 × 10^4^ per well) into 96-well plates in triplicates and allowed to adhere overnight. On the following day, the medium was removed, and fresh medium alone or containing the unconjugated mAbs (1 μg/ml), ATC (2 x10^5^/well), HER2Bi-armed ATC (2 x10^5^/well, armed with 50 ng/HER2Bi/10^6^ ATC) or a combination of OKT3 and Herceptin® with ATC (unarmed ATC). Cultures was incubated for 18 hours, then floating was removed and 100 µl of fresh medium containing 1/10 (v/v) Cell Counting Kit-8 (CCK8, Dojindo Laboratories, Kumamoto, Japan) reagent was added to each well and incubated for an additional 3 hours. After incubation, the absorbency of melanoma cell was measured using a 96-well plate reader (DG5032, Huadong, Nanjing, China) at 450 nm. Wells containing the CCK-8 reagent but no cells were used as the blank control. Cell proliferation was assessed by the absorbance values according to the manufacturer’s protocol.

### 7: Flow cytometry analysis

To detect the expression of HER2 on the cell surface, melanoma cells (10^6^) were incubated for 30 minutes on ice with Herceptin®, followed by staining with FITC-labeled goat-anti-human-IgG. To detect HER2Bi-Ab bound to HER2^+^ or CD3^+^ cell, Malme-3M-luc or ATC were incubated with HER2Bi-Ab (1 μg /ml) or a combination of OKT3 and Herceptin® (1 μg/ml, used as the negative control for HER2Bi-Ab) for 30mins; bound HER2 moiety of HER2Bi-Ab to Malme-3M-luc cells was evaluated using FITC-labeled goat anti-mouse IgG to detect the anti-CD3 moiety of the Bi-Ab; bound CD3 moiety of HER2Bi to ATC was evaluated by using FITC-labeled goat-anti-human-IgG to detect the HER2 moiety of the Bi-Ab. To detect the CD69 expression on ATC, the floating cells from Malme-3M and ATC co-culture were incubated with anti-CD69-PE and anti-CD3-FITC (UCHT1, eBioscience). The cells were washed three times and assayed with a Guava flow cytometrometer Easycyte (Guava Technologies, Hayward, CA) and the data were analyzed using FlowJo software (Tree Star, Ashland, OR, USA). The mean florescence intensity (MFI) was calculated by subtracting MFI value stained with control IgG from the cells stained with specific Ab.

### 8: Western blot

For western blot analysis, the cells (10^6^) were lysed and centrifuged, and the supernatants were boiled in SDS sample buffer for 5 minutes and separated by SDS-PAGE in a 12% polyacrylamide gel. The proteins in the gel were then transferred to Hybond C Extra membrane (Amersham) at 20 V for 2 hours. The membrane was blocked in 10 ml of PBS containing 2% Marvel milk (Premier International Foods, St Albans, UK) on a plate rocker (Stuart Gyro Rocker STR-9) for 1 hour and transferred to 1:2500 Herceptin® or anti-β-actin mAb (Zhongshan Golden Bridge, China) in 10 ml of PBST supplemented with 2% Marvel milk and incubated at 4°C overnight. On the next day the membrane was washed 3 times and incubated with 10 ml of PBST/2% Marvel milk containing 1:5000 goat anti-human IgG-HRP conjugate or goat anti-mouse IgG (1:10000, Zhongshan golden bridge). The washed membrane was developed in an ECL kit and exposed to Kodak Biomax film for 5 minutes.

### 9: In vitro cytotoxicity assay

Cytotoxicity of T cells against target cells was measured with a luciferase quantitative assay [28,29]. Briefly, Malme-3M-luc cells or B16-luc cells were seeded (10^4^ /well) into 96-well round bottom microplate in triplicates overnight. On the following day, the medium was removed, and fresh medium alone or containing HER2Bi-armed ATC or unarmed ATC was added to wells at various effector-to-target ratios (E/T) of 1:1, 5:1, and 20:1. ATC and target Malme-3M-luc cells were incubated at 37°C for 18 hours. A final concentration of 0.15 mg/ml luciferin (gold bio) was added to each well, and the bioluminescence image signal was measured with the Xenogen IVIS system. The specific cytotoxicity by HER2Bi-armed ATC was determined in photons per second.

### 10: IFN-γ ELISA

Malme-3M-luc cells were seeded (10^4^ /well) into round bottom 96-well microplates in triplicates overnight. The medium was removed, and fresh medium or medium containing HER2Bi-armed ATC or unarmed ATC was added to wells at various E/T of 1:1, 5:1, and 20:1. ATC and target Malme-3M-luc cells were incubated at 37°C for 18 hours. Then the supernatants were collected and IFN-γ was quantified by a human IFN-γ ELISA kit (Thermo Scientific) according to the manufacturer’s instructions. The plate was read in an ELISA reader (ELx808, Bio-Tek Instruments Inc., Houston, TX) with a 450 nm filter. The absorbance obtained from the standards was plotted and values were calculated.

### 11: In vivo antitumor effect of HER2Bi-armed ATC in mouse model

Malme-3M-luc cells (1 x10^6^ /mouse) alone or were mixed with unarmed ATC (250 ng Herceptin® and OKT3, combined with 1 x10^7^ATC/mouse), or HER2Bi-armed ATC (1 x10^7^ /mouse, armed with 50 ng/HER2Bi/10^6^ ATC) in 200 μl PBS. The cell mixtures were immediately inoculated subcutaneously on the rear flank of SCID-Beige mice (Vital River Laboratory Animal Technology Co. Ltd, Beijing). In order to accurate follow up of tumor growth, in vivo bioluminescence imaging was operated on day 2, 7 and 14 after tumor inoculation. Bioluminescent imaging was done using Xenogen IVIS-100 imaging system with living image software (Caliper Life Science). Briefly, mice were injected by i.p. with D-luciferin (15 mg/ml) suspended in 100 μl PBS and imaged under pentobarbital sodium (6 mg/ml, Sigma) anesthesia after 10 minutes. Exposure conditions (time, aperture, stage position, binning, and time after injection) were kept identical in all measurements. All mice studies were carried out under a protocol approved by the Institutional Animal Care and Use Committee.

### 12: Statistical analyses and reproducibility

All experiments were repeated at least twice and mostly three times. Statistical analyses were performed by independent *t*-test or one-way analysis of variance (ANOVA) followed by LSD for multiple comparison. *P* < 0.05 was considered significant compared with a control group.

## Results

### 1: Confirmation of HER2 expression in human melanoma cells

**Figure 1:**
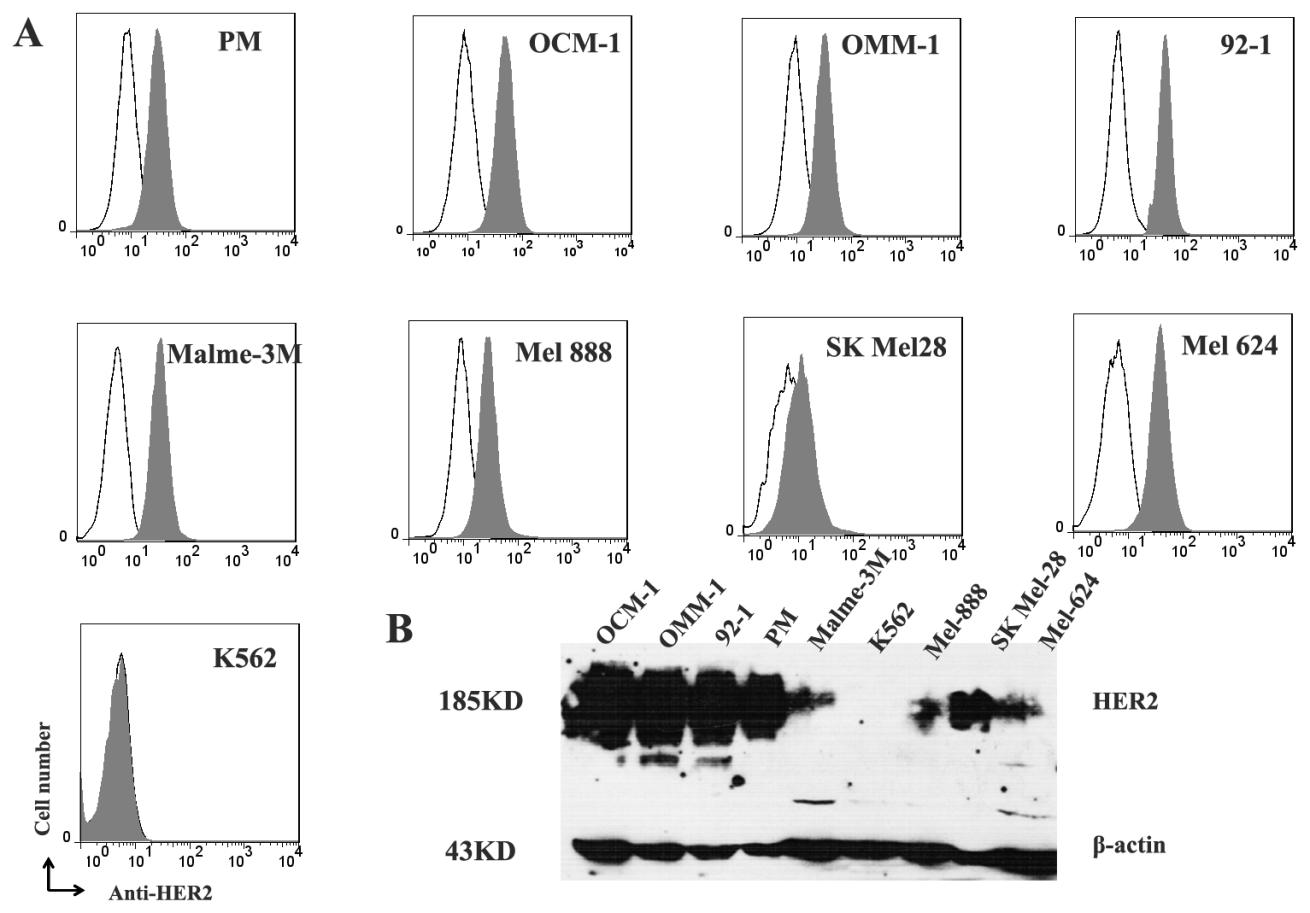
Expression of HER2 in different human melanoma cells. (A) Surface expression of HER2 was evaluated by flow cytometry on seven melanoma cell lines (OCM-1, OMM-1, 92-1, Malme-3M, Mel888, SK Mel28 cell, and Mel624) and a primary melanoma (PM) cell. Shaded histograms represent cells stained with Herceptin® and open histograms represent cells stained with a non-related human IgG as control. (B) Western blot analysis showed HER2 protein band (185 kD) in cell lysates of human melanoma cells. β-actin was used as an internal control. A HER2 non-expression cell line K562 was used as a negative control.

The surface expression of HER2 on human melanoma cells was assessed by FACS analysis. As shown in Figure 1A, HER2 expression was detected on all the human melanoma cells including the primary culture and OCM-1, 92-1, OMM-1, Malme-3M, Mel624, Mel888, and SK Mel28. In contrast, HER2 expression was not observed on K562 cells, which was used as a negative control.

Western blot confirmed that HER2 protein presented in the cell lysates of all human melanoma cells, on the contrary, no HER2 band was detected in the lysate of K562 cells. Interestingly, HER2 expression in uveal melanoma cell line showed much stronger than that in skin melanoma cell line, suggesting the intracellular expression of HER2 by uveal melanoma cell.

### 2: Establishment of a stable Malme-3M cell line that expressed firefly luciferase

**Figure 2:**
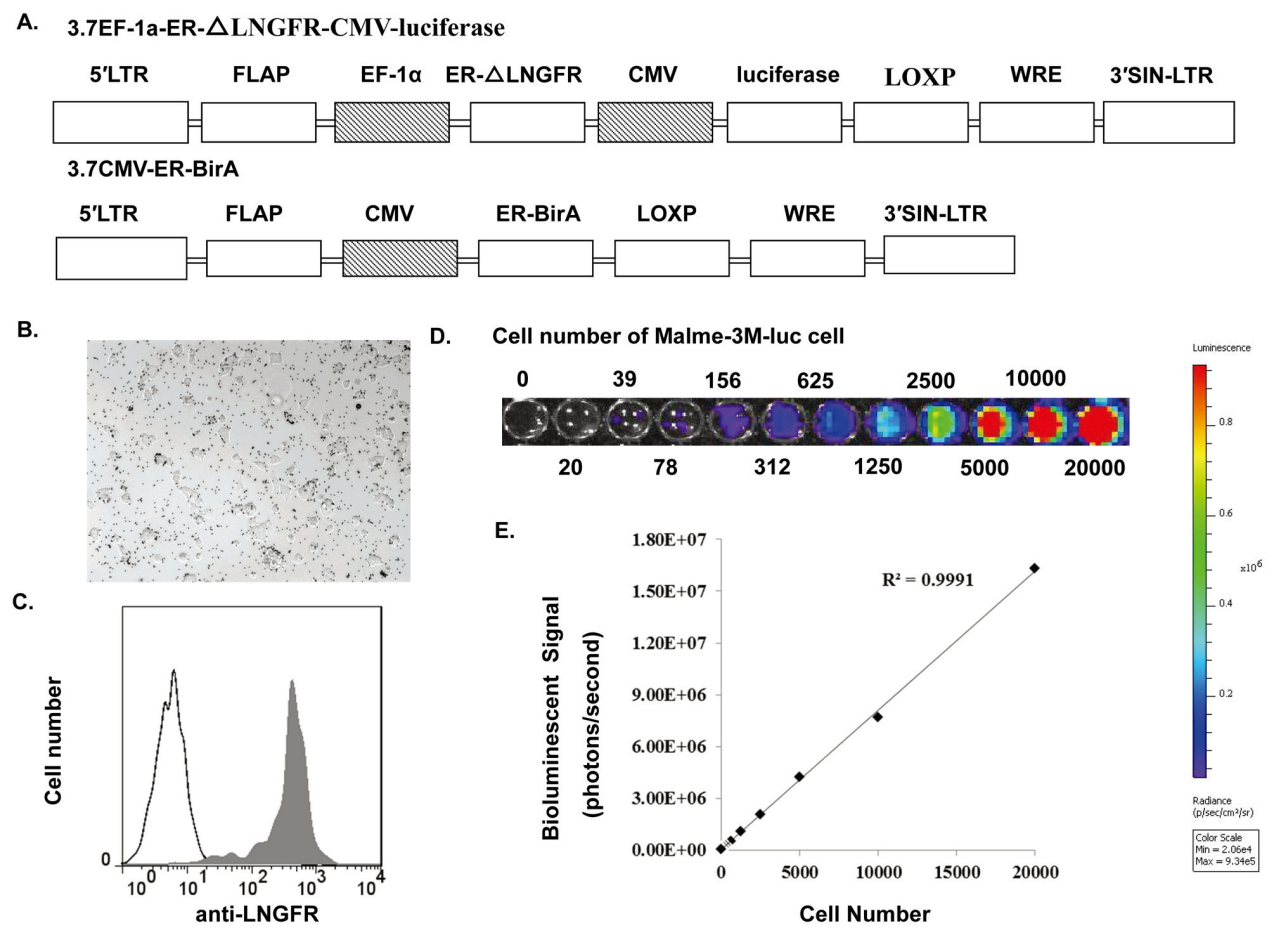
Generation of Malme-3M-luc cells. (A) Schematic representation of the lentivirus vectors 3.7EF-1α-ER-△LNGFR-CMV-luciferase and 3.7CMV-ER-BirA. (B) Malme-3M cells were transduced with both 3.7lnluc and 3.7BirA lentiviral vectors and luc-positive cells were isolated by streptavidin beads. (C) FACS analysis of LNGFR expression in isolated Malme-3M-luc cells. Cells were labeled with anti-LNGFR monoclonal antibody CD271 followed by goat-anti-mouse-PE-CY5. Histogram indicates LNGFR expression, in which the shaded histogram represents purified Malme-3M-luc cells, and the black line represents un-transduced Malme-3M cells. (D) The bioluminescence image signal was measured with the Xenogen IVIS system, and serial dilution of Malme-3M-luc cells were plated in black 96-well plate in PBS containing D-luciferin substrate at final concentration of 0.15 mg/ml. (E) Pearson’s correlation coefficient analysis. The correlation coefficient (R) between the luciferase quantity and living cell number is 0.999.

To assess tumor growth and therapeutic effect over time, we generated a stable Malme-3M cell line that expressed a firefly luciferase reporter. To facilitate the establishment of the stable cell line, we employed a novel lentiviral vector system (Figure 2A). This system could label genetically modified cells with a cell surface biotinylation tag by co-transfecting cells with BirA, a biotin ligase. The modified cells could be quickly isolated for the downstream applications using a simple *luciferase* method. For this study, we constructed two lentiviral vectors. The first one was 3.7lnluc, containing a firefly luciferase reporter gene and a BirA-tag, and the second one was 3.7cmvBirA containing BirA enzyme for catalysing biotinylation of BirA tag. Two vectors were co-transduced into Malme-3M cells, and cells were selected for stable luciferase expression using streptavidin beads (Figure 2B). The majority of luciferase-positive cell clones were repeatedly selected by limiting dilution. One of the clones expressing both luciferase and LNGFR-BirA-tag was detected by FACS staining with an anti-LNGFR antibody (Figure 2C). The clone was chosen for the subsequent studies and designated as Malme-3M-luc. To determine luciferase activity from the established cell line, we seeded Malme-3M-luc cells into 96-well plates at different concentrations, ranging from 20000 to 20 (20000, 10000, 5000, 2500, 1250, 625, 312, 156, 78, 39, 20) cells per well. Luciferase activity was quantitatively analyzed using a Xenogene IVIS-100 instrument along with live image software. The results showed that there was a good correlation between the level of luciferase activity and the number of cells seeded (Figure 2D). In Figure 2E, the linear relationship of luciferase-dependent photon production and serially diluted Malme-3M-luc cells was shown (r^2^=0.999, p<0.0001).

### 3: Luciferase gene did not affect HER2 expression and the proliferation of Malme-3M cells

**Figure 3:**
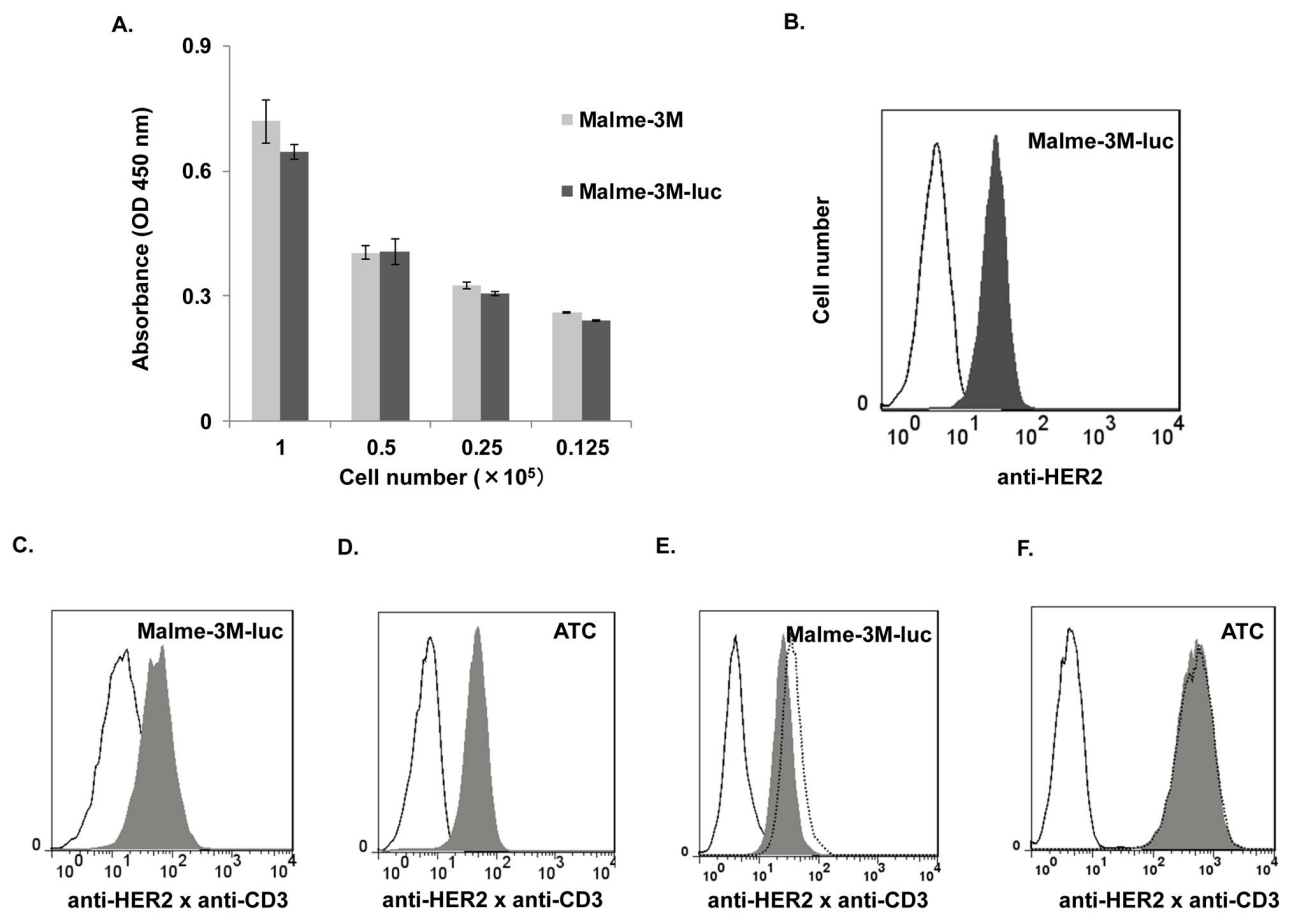
No effect of the luciferase gene on HER2 expression and the proliferation of Malme-3M cells and the binding of anti-CD3×anti-Her2 Bispecific antibody (HER2Bi-Ab) to both HER2 positive and CD3 positive cells. (A) Proliferation of Malme-3M and Malme-3M-luc cells was assessed by CCK8 assay. The experiments were triplicated and the data are shown as the mean absorbance with SD (at OD 450 nm). Statistical analysis was conducted using Student’s *t*-test. (B) Surface expression of HER2 on Malme-3M-luc cells was analyzed by flow cytometry. Shaded histogram represents cells stained with Herceptin®, and open histogram represents cells stained with a control human IgG. (C) Malme-3M-luc cells were incubated with HER2Bi-Ab (1μg/ml, shaded histogram) or a combination of OKT3 and Herceptin® (1 μg/ml, open histogram) for 30 minutes at room temperature. HER2Bi-Ab binding was analyzed by flow cytometry using FITC- goat-anti-mouse IgG to detect the anti-CD3 moiety of the HER2Bi-Ab. (D) Activated T cells (ATC) were incubated with HER2Bi-Ab (1μg/ml, shaded histogram) or a combination of OKT3 and Herceptin® (1 μg/ml, open histogram) for 30 minutes at room temperature. HER2Bi-Ab binding was analyzed by flow cytometry using FITC- goat-anti-human IgG to detect the anti-HER2 moiety of the HER2Bi-Ab. (E) Malme-3M-luc cells were incubated with HER2Bi-Ab (1μg/ml, shaded histogram, OKT3 (1μg/ml, open histogram), or Herceptin® (1μg/ml, dot histogram) for 30 minutes at room temperature. HER2Bi-Ab binding was analyzed by flow cytometry using FITC- goat-anti-human IgG to detect the anti-HER2 moiety of the HER2Bi-Ab. (F) ATC were incubated with HER2Bi-Ab (1μg/ml, shaded histogram), Herceptin® (1μg/ml, open histogram), or OKT3 (1μg/ml, dot histogram) for 30 minutes at room temperature. HER2Bi-Ab binding was analyzed by flow cytometry using FITC- goat-anti-mouse IgG to detect the anti-CD3 moiety of the HER2Bi-Ab.

To investigate whether bio-photonic activity or the luciferase gene itself had a negative influence on Malme-3M cell growth in vitro, proliferation assays were performed (Figure 3A) as described in the materials and methods. To measure the proliferation of cells stably expressing luciferase, the proliferation rates of Malme-3M and Malme-3M-luc cells were assessed by a CCK8 assay. No significant difference in cell viability between Malme-3M cells and Malme-3M-luc cells was observed, which revealed that luciferase gene did not alter the proliferation of cells as previously reported by Tiffen [30]. Furthermore, the expression level of HER2 on Malme-3M cells and Malme-3M-luc cells was similar (Figure 3B). Taken together, these data indicated that luciferase gene did not affect the proliferation and HER2 expression of Malme-3M cells.

### 4: Preparation and characterization of HER2Bi-Ab

To evaluate the ability of HER2Bi-Ab to bind to the target Malme-3M-luc cells, Malme-3M-luc cells were incubated with HER2Bi-Ab or a combination of OKT3 and Herceptin®, used as the negative control. The binding of HER2Bi-Ab was evaluated by flow cytometry using FITC goat-anti-mouse IgG to detect the anti-CD3 moiety of the HER2Bi-Ab. As shown in Figure 3C, positive stained cells were detected in 70% of the Malme-3M-luc population with a mean fluorescent intensity of 80, as nearly the same MFI as Herceptin® staining; in contrast, using FITC goat-anti-mouse IgG, the combinations of OKT3 and Herceptin® were not detected to bind to Malme-3M-luc cells. Moreover, binding of HER2Bi-Ab to Malme-3M-luc cells was confirmed by flow cytometry using FITC goat-anti-human IgG to detect the anti-HER2 moiety of the HER2Bi-Ab (Figure 3E)

To evaluate the binding of HER2Bi-Ab to ATC, ATC were incubated with HER2Bi-Ab or a combination of OKT3 and Herceptin® as a negative control for HER2Bi-Ab. HER2Bi-Ab binding was confirmed by flow cytometry using FITC goat-anti-human IgG to detect the anti-HER2 moiety of the HER2Bi-Ab. Positive stained cells were detected in 97% of the ATC population with a mean fluorescent intensity of 50 (Figure 3D); in contrast, using FITC goat-anti-human IgG for negative control OKT3 and Herceptin® staining, there was no antibody detected to bind to ATC. Moreover, binding of HER2Bi-Ab to ATC was confirmed by flow cytometry using FITC goat-anti-mouse IgG to detect the anti-CD3 moiety of the HER2Bi-Ab (Figure 3F).

### 5: Growth inhibition of melanoma cells by HER2Bi-armed ATC

**Figure 4:**
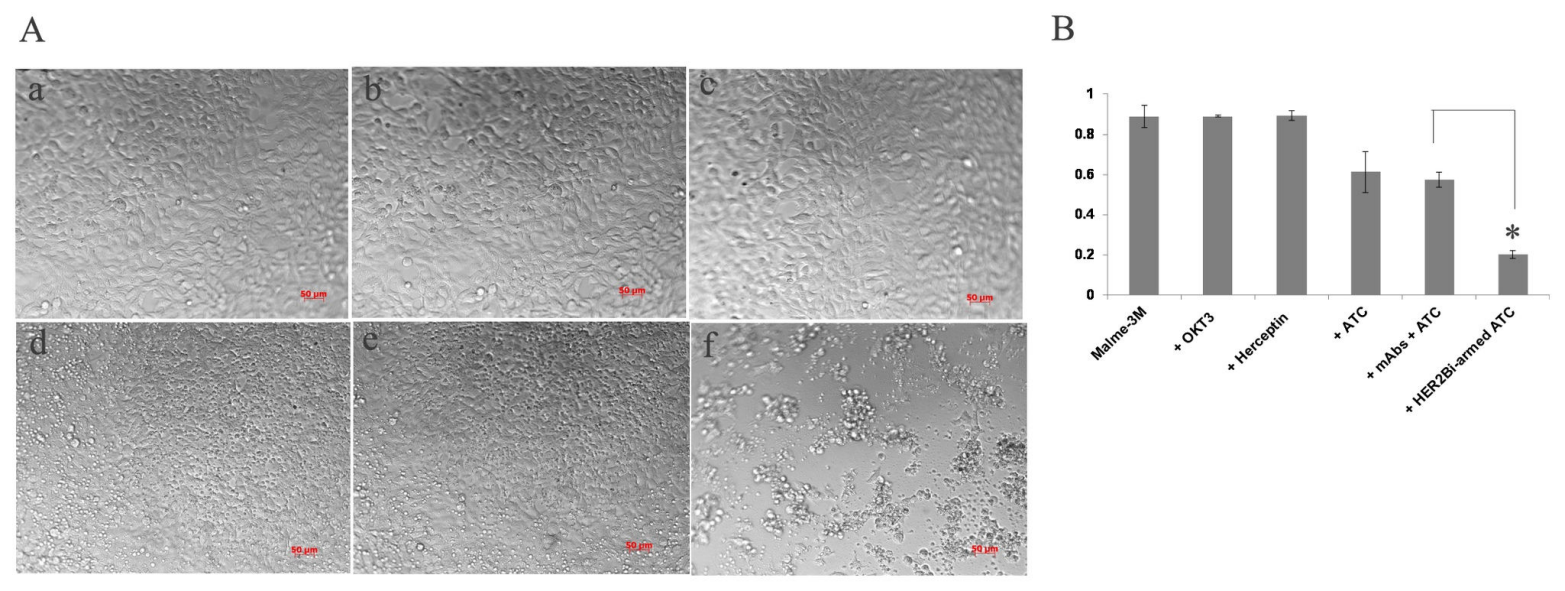
Growth inhibition of melanoma cells by HER2Bi-armed ATC. Malme-3M-luc cells were seeded (2 x10^4^/well) into 96-well round bottom microplate in triplicates overnight. On the following day, the medium was removed, and fresh medium alone or containing the unconjugated mAbs OKT3 or Herceptin (1μg/ml), ATC (2 x10^5^/well), HER2Bi-armed ATC (2 x10^5^/well, armed with 50 ng/HER2Bi/10^6^ ATC) or a combination of OKT3 and Herceptin® with ATC (unarmed ATC) for 18 hours. (A) Real-time photographs were taken at 200x magnification. a. Malme-3M-luc targets alone; b. Malme-3M-luc with OKT3; c. Malme-3M-luc with Herceptin; d. Malme-3M-luc with ATC; e. Malme-3M-luc with unarmed-ATC; f. Malme-3M-luc with HER2Bi-armed ATC. HER2Bi-armed ATC, but not ATC or unarmed ATC aggregated with Malme-3M-luc tumor cells in culture after 18 hours incubation. (B) The proliferation of Malme-3M-luc was assessed by CCK8 assay with the OD value measured at 450 nm. The data are mean ± SD of triplicate experiments, and a representative experiment of three was shown. Statistical analysis was conducted using Student’s *t*-test. *: *P*<0.01, HER2Bi-armed ATC compared with combination of the OKT3 and Herceptin with ATC under similar conditions.

The amount of HER2Bi required to arm ATC was ranging from 5 to 500 ng/10^6^ cells. Since 50 ng and 500 ng/10^6^ cells showed a similar cytotoxicity, we chose 50 ng/10^6^ cells as the concentration of HER2Bi for all subsequent experiments. In cell proliferation assays, unconjugated mAbs (OKT3 or Herceptin®), ATC alone, a combination of OKT3 and Herceptin® with ATC (unarmed ATC), or HER2Bi-armed ATC (E/T of 10:1) were co-cultured with Malme-3M-luc cells for 18 hours, respectively. Real-time photographs of each group were taken at 200x magnification. It was found that HER2Bi-armed ATC, but not ATC or unarmed ATC aggregated with Malme-3M-luc cells in culture after 18 hours incubation (Figure 4A). Then the floating was removed, and 100 µl of fresh medium containing CCK8 reagent was added to each well and incubated for an additional 3 hours. The HER2Bi-armed ATC showed a superior growth inhibition of target cells compared to the other test groups (Figure 4B).

### 6: Cytotoxity effects on melanoma cells of HER2Bi-armed ATC with IFN-γ production

**Figure 5:**
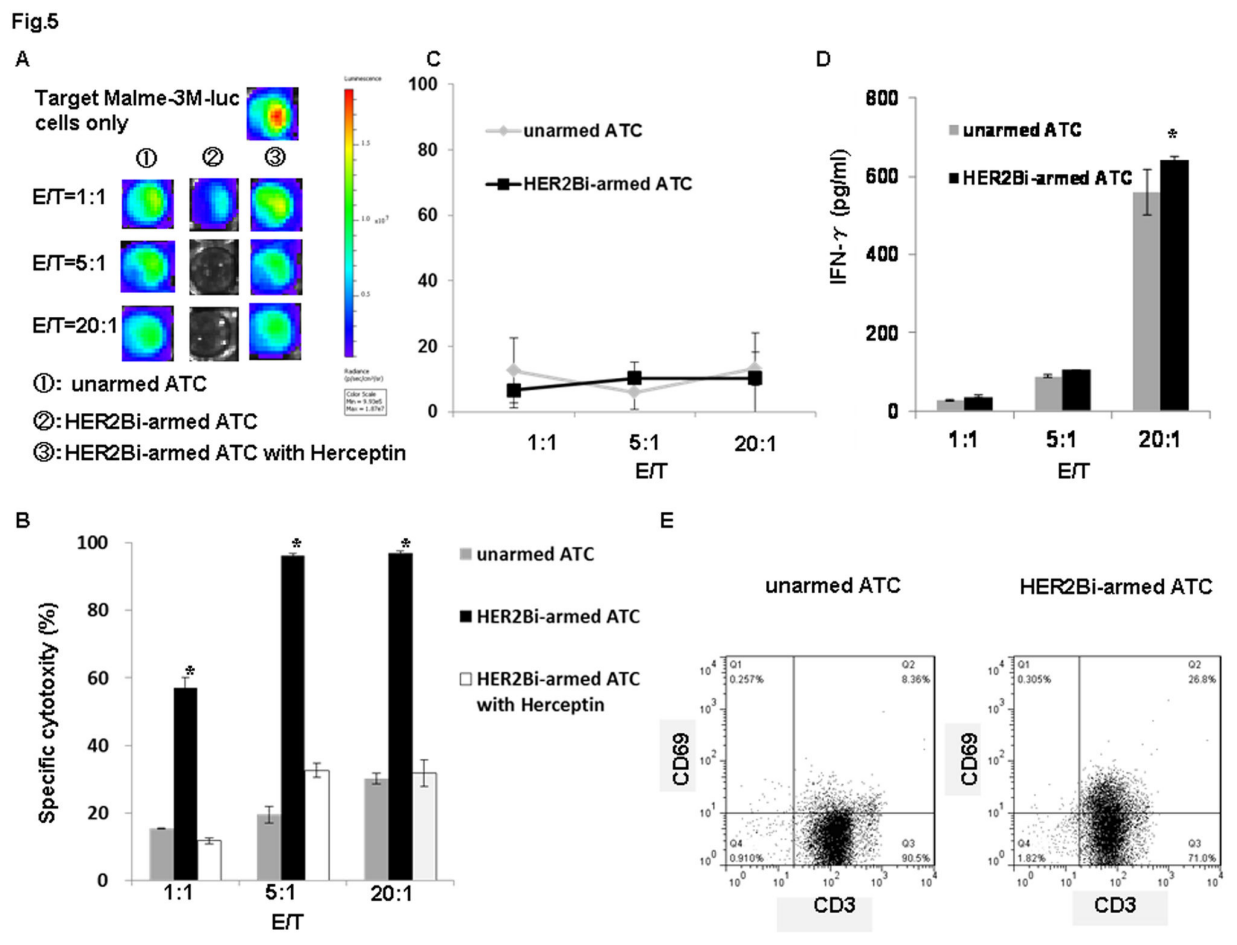
The lytic activity of HER2Bi-armed ATC against Malme-3M-luc cells (A) Bioluminescence images of Malme-3M-luc cells after incubation with HER2Bi-armed ATC or unarmed ATC at different E/T ratio (1:1, 5:1, and 20:1) for 18 hours. (B) Bioluminescence image signal in photons per second was converted into living cell number and the cytotoxicity assay was measured at the indicated E/T. (C) The lytic activity of HER2Bi-armed ATC against B16-luc cells. (D) IFN-γ secretion by HER2Bi-armed ATC against Malme-3M–luc cells. Supernatants of co-cultures at indicated E/T were harvested at 18 hours and analyzed for IFN-γ level using IFN-γ ELISA Kit. (E) Expression of CD69 on HER2Bi-armed ATC or unarmed ATC was detected by flow cytometry after 18 hours co-culture with Malme-3M-luc cell at E/T ratio of 5:1. For B and C, the data are mean ± SD of triplicate experiments. Statistical analysis was conducted using Student’s *t*-test. A representative experiment of at least two was shown. *: *P*<0.01, HER2Bi-armed ATC compared with unarmed ATC under similar conditions.

Cytotoxicity assays were performed at E/T ratios of 1:1, 5:1 and 20:1 for 18 hours. The bioluminescence images correlated with the number of living Malme-3M-luc cells as shown in Figure 5A. After 18 hour incubation with HER2Bi-armed ATC or unarmed ATC, bioluminescence image signal expressed in photons per second was converted into living cell number and the cytotoxicity assays was calculated at the indicated E/T ratios. As shown in the Figure 5B, increasing E/T ratio correlated directly with the percentage of cytotoxicity in both armed-ATC and unarmed-ATC effectors. The percentage of cytotoxicity of armed ATC was significantly greater than that of unarmed effectors at different E/T ratios. Moreover, the HER2Bi-armed ATC mediated cytolysis was blocked by the addition of Herceptin, which confirmed the HER2-specific killing. On the contrary, there was no difference in the cytotoxicity against mouse melanoma cell line B16-luc between HER2Bi-armed ATC and unarmed ATC (Figure 5C).

To analyze the cytokines involved in cytotoxicity, **c**ell culture supernatants were investigated for IFN-γ production. As shown in Figure 5D, the IFN-γ production increased with the enhanced E/T ratio in both unarmed and HER2Bi-armed ATC as effectors, with the concentration ranging from 27 to 641 ng/ml. Although no significant difference was found at E/T ratios 1: 1 and 5: 1, a remarkable increase was observed in IFN-γ secretion in the supernatants from HER2Bi-armed ATC over their unarmed ATC counterpart when ATC were co-cultured with Malme-3M-luc cells at a E/T ratio 20:1（p<0.01).

Moreover, FACS analysis of HER2Bi-armed ATC showed increased CD69 expression over their unarmed ATC counterpart after 18 hour incubation with target cells, 26.8% versus 8. 36%, respectively (Figure 5E).

### 7: HER2Bi-armed ATC prevented Malme-3M tumor growth in SCID-Beige mice

**Figure 6:**
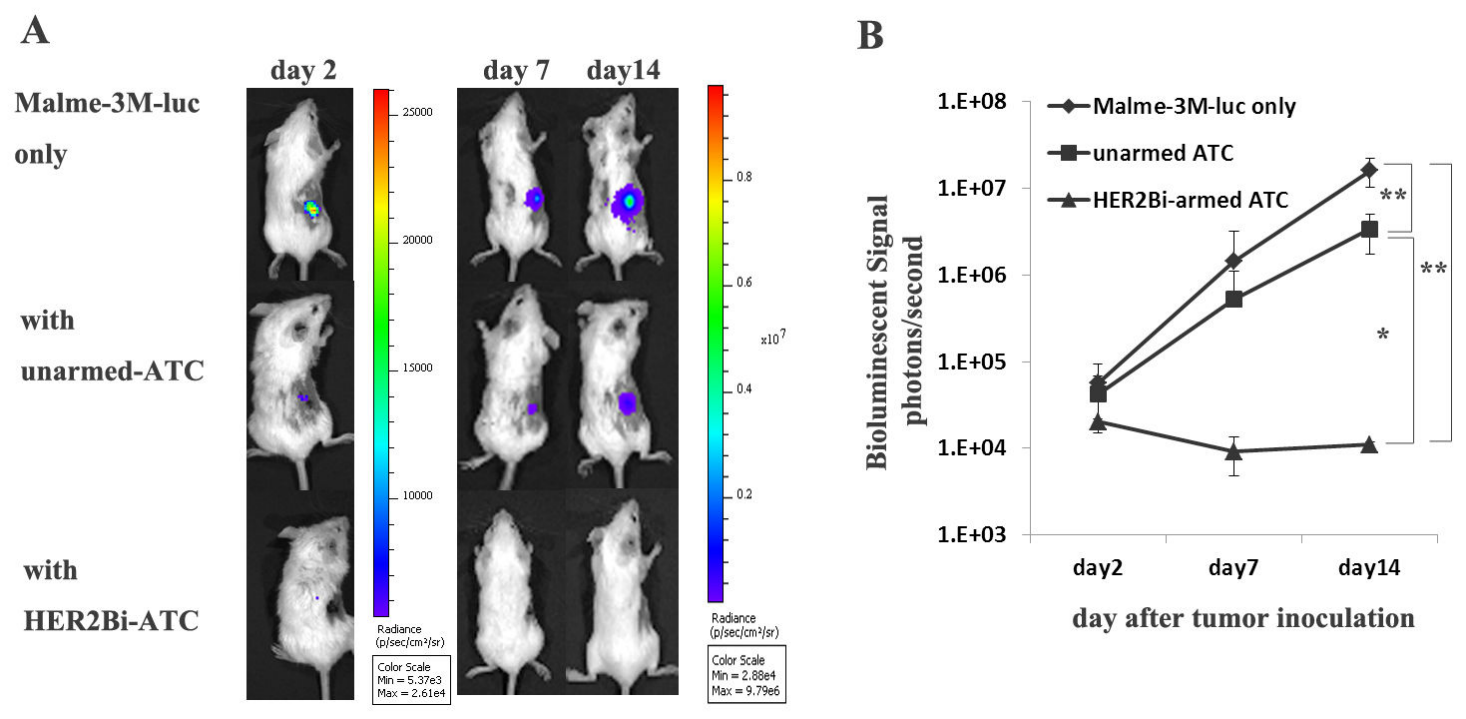
In vivo cytotoxicity of HER2Bi-armed ATC against Malme-3M-luc cells. SCID-Beige mice were inoculated with Malme-3M-luc cells (1 x10^6^/mouse) alone, or a mixture of Malme-3M-luc cells with un-armed ATC (250 ng Herceptin® and OKT3, combined with 1 x10^7^ATC/mouse), or with HER2Bi-ATC (1 x10^7^ /mouse, armed with 50 ng/HER2Bi/10^6^ ATC) on day 0. Each group contained 5 mice. (A) Bioluminescence images of a representative mouse from each group on day 2, 7 and 14 were shown. (B) Images were analyzed using Living Image software and tumor values were represented as total flux measurements in photons/second. Mean values of tumor growth curves were shown. The significance between different groups was compared by one-way analysis of variance (ANOVA) followed by LSD for multiple comparison. **P<0.01, *P<0.05.

To further determine whether HER2Bi-armed ATC could suppress tumor growth in vivo, SCID-Beige mice were engrafted subcutaneously with Malme-3M-luc cells. The mice were treated with unarmed ATC or HER2Bi-ATC, respectively. The growth of tumor was monitored with bioluminescent imaging. This bioluminescent imaging model allows of monitoring tumor cell fate as early as the first few days after inoculation, when tumor formation cannot be detected by palpation. In Figure 6A, one representative mouse of each group was shown. When Malme-3M-luc cells were inoculated alone, strong light signal was observed on day 2 and increased over time till day 14. A similar, but slower, weaker kinetics of tumor growth was shown in mice that were co-injected with unarmed-ATC. However, when Malme-3M-luc cells were co-injected with HER2Bi-armed ATC, the signal almost disappeared on day 2 and vanished completely from day 7 to day 14. The mean bioluminescence signal of each test group correlated with the number of living Malme-3M-luc cells as shown in Figure 6B. In mice treated with either HER2Bi-armed ATC or unarmed ATC, the tumor growth was significantly inhibited compared with mice treated with Malme-3M-luc alone. Moreover, the inhibitory effect of HER2Bi-armed ATC was superior to that of unarmed ATC.

## Discussion

In this study, we examined HER2 expression in human melanoma cells and took advantage of luciferase as a bioluminescence reporter for cell viability to establish Malme-3M-luc cell line with stable and high level luciferase activity. The present study revealed several new findings relevant to the therapeutic application of targeting HER2 against melanoma. Firstly, the expression of HER2 in several melanoma cell lines was confirmed by FACS analysis and western blot detection. In vitro cytotoxicity assay showed that anti-HER2 antibody had no inhibitory effect on melanoma cell lines, but HER2Bi-armed ATC provided significant anti-proliferative and cytotoxic activity against Malme-3M cells. Moreover, HER2Bi-armed ATC secreted significantly higher level of IFN-γ than unarmed ATC counterpart against Malme-3M-luc cells.

The finding of HER2 expression in this study was in line with earlier studies in cell lines [22,23] and clinical specimens [24] of primary and metastatic malignant melanoma, whereas contradicted with some other studies in clinical specimens of metastatic malignant melanoma [17–21]. The reason for the lack of concordance between these studies is still not clear. It is probably due to individual differences of patients or the way the tissue was processed or different sensitivities of the assays used. In addition, the level of HER2 expression may also be a contributing factor for determination of HER2 presence in melanoma by different groups. In this study, HER2 expression was detected in cell culture from both skin and uveal melanoma. The full-length HER2 is a 185 kDa protein, and sometimes, a soluble truncated HER2 may arise mainly through a slow proteolytic cleavage in HER2^+^ tumor cells in culture as reported in [31]. Moreover, HER2 expression in uveal melanoma cell line was much stronger than that in skin melanoma cell line, suggesting the intracellular expression of HER2 in uveal melanoma cell.

To efficiently analyze effector’s activity to a target tumor cell, we established a Malme-3M-luc cell line stably expressing a high level of luciferase. As our data shown, the luciferase gene did not affect the expression of HER2 and the proliferation of Malme-3M cells, which was in line with the study of Tiffen group [30]. Importantly, there was a good correlation between the level of luciferase activity and the number of living Malme-3M cells, therefore, in our experiment, cytotoxicity was successfully measured using a luciferase quantitative assay. We also generated a xenograft mouse model for human melanoma using Malme-3M-luc cells, and the xenograft mouse supplied a sensitive model for monitoring and treating human melanoma in vivo.

The anti-tumor effect of Herceptin® on breast cancer was reported to involve three main mechanisms: direct inhibition against cell growth, antibody-dependent cell-mediated cytotoxicity (ADCC) and T-cell mediated lysis. The induction of T-cell-secreted cytokines, e.g. TNF-α and IFN-γ, further supported its anti-tumor effect [32]. However, even with efficient blockade of oncogenic signals and induction of proper signals against tumor cells, antibody-initiated immunity may be transient and weak, making further combination immunotherapy necessary to reach clinical significance [33]. One approach is to recruit and augment the patient’s own immune system to target tumor cells by redirecting ex vivo expanded anti-CD3 activated T cells with a bispecific antibody. Treatment of eight melanoma cells in our study with Herceptin® did not affect the growth rate as determined by CCK-8 assay (data not shown), which were probably due to the relatively weak expression of HER2 in melanoma and the absence of FcR^+^ cells in vitro, for that FcR^+^ cells were shown to be essential for mediating the therapeutic effects of HER2/neu antibody [34]. Our study showed that HER2Bi-armed ATC had significant anti-proliferative and cytotoxic activity against Malme-3M cells. Moreover, HER2Bi-armed ATC expressed higher level of activation marker CD69 and secreted significantly higher level of IFN-γ than unarmed ATC counterpart against Malme-3M-luc cells. Therefore, T-cell cytotoxicity is dependent upon the engagement of the HER2 tumor-associated antigen via HER2Bi-Ab bridge. Arming leads to not only binding specifically to tumor cells but also triggering T cell activation and cytokine secretion. Our data from in vitro cytotoxicity assays indicated HER2Bi-armed ATC mediated cell killing without the addition of IL-2 to the cultures. HER2Bi-armed ATC are currently under investigation in phase I/II clinical trials for breast cancer and immune evaluation studies of these patients are ongoing in an effort to gain further insights into the immunostimulatory effects of this therapy [35]. In our experiment, although the expression of HER2 in melanoma cells was not very strong, HER2Bi-armed ATC showed significant anti-proliferative and cytotoxic activity, which indicated that development of bispecific antibodies would be benefit in the field of targeting therapeutics. Arming of ATC with bispecific antibodies engineered to bind CD3 on one end and the specific tumor antigen on the other end is a feasible approach for overcoming the practical limitations in cancer immunotherapy with regard to specificity and magnitude of antitumor T-cell populations.

To conclude, we have demonstrated HER2 expression in melanoma cells and potent melanoma cell killing by HER2Bi-armed ATC. Future efforts will be needed to evaluate the effects of HER2Bi-armed ATC on human melanoma in vivo in detail. Our findings provide further support for the potential benefit of HER2Bi-armed ATC for treatment of melanoma.
