# Correction: HER2 as a Promising Target for Cytotoxicity T Cells in Human Melanoma Therapy

**DOI:** 10.1371/annotation/611f44b7-e349-4e7f-9dfe-bdfc724c2f4c

**Published:** 2013-09-05

**Authors:** Juan Ma, Huamin Han, Deruo Liu, Wei li, Hongxiang Feng, Xin Xue, Xiaoran Wu, Ge Niu, Ge Zhang, Yunfeng Zhao, Changzhen Liu, Hua Tao, Bin Gao

There was an error in the Funding statement. The correct version of the Funding statement is available below.

This work is funded by the grants from the Ministry of Science and Technology of China (S & T major Program: No. 2012ZX1004701-001-002), the Basic Research Program of China (973 Program, No. CB531502), the National Nature Science Foundation of China (No. 31170829, 31070783, 81021003), and Beijing Natural Science Foundation (No. 5112022). The funders had no role in study design, data collection and analysis, decision to publish, or preparation of the manuscript. 

